# Aortic valve replacement of a quadricuspid aortic valve with right coronary artery ostium adjacent to one of the commissures

**DOI:** 10.1186/s13019-022-01900-z

**Published:** 2022-06-07

**Authors:** Shigeto Tsuji, Shogo Shimada, Yoshifumi Itoda, Haruo Yamauchi, Minoru Ono

**Affiliations:** 1 Department of Cardiac Surgery, The University of Tokyo, 7-3-1, Hongo, Bunkyo-ku, Tokyo, 113-8655 Japan; 2 Department of Cardiovascular Surgery, IMS Katsushika Heart Center, 3-30-1, Horikiri, Katsushika-ku, Tokyo, 124-0006 Japan

**Keywords:** Quadricuspid aortic valve, Aortic regurgitation, Aortic valve replacement, Coronary ostium anomaly, Non-everting mattress fashion, Complete atrioventricular block

## Abstract

**Background:**

Quadricuspid aortic valve is a rare congenital heart disease that may be associated with a different anatomical relationship between the coronary artery ostium and the commissure.

**Case presentation:**

Herein, we report a case of a 59-year-old woman who underwent aortic valve replacement for a quadricuspid aortic valve with severe aortic regurgitation. Intraoperatively, the aortic valve had four cusps of almost equal size and the right coronary artery arose adjacent to the commissure between the right coronary cusp and one of the two non-coronary cusps. The annular stitches were placed in a non-everting mattress fashion with pledgets on the ventricular side, and stitches near the right coronary ostium were transitioned to the subannular ventricular myocardium to maintain the distance from the ostium. A one-step smaller-sized prosthesis was selected to avoid an oversized prosthetic valve potentially compressing the right coronary ostium.

**Conclusions:**

When performing aortic valve replacement for a quadricuspid aortic valve, careful observation of the coronary location and means to avoid coronary ostium obstruction are essential.

## Background

Quadricuspid aortic valve (QAV) is a rare congenital heart disease that is less common than bicuspid and unicuspid aortic valve [1–5]. Coronary artery and coronary ostium anomalies are present in 2–10% of patients with QAV [2, 3, 6–8]. The functional status of QAV is predominantly pure aortic regurgitation (AR) [3, 6]. Clinical manifestations, such as palpitations, dyspnea, fatigue, and chest pain, depend on the functional status of QAV and usually present in the fifth or sixth decade of life [2]. Standard surgical repair is through aortic valve replacement (AVR), although aortic valve plasty, such as bicuspidalization and tricuspidalization, is also performed [6]. We performed AVR for a QAV that was associated with the right coronary artery ostium adjacent to one of the commissures. We describe a modification of the annular stitches to prevent right coronary ostium obstruction.

## Case presentation

A 59-year-old female patient with asymptomatic severe AR was referred to our institution for surgical treatment. She was being followed up at an outpatient clinic for QAV and moderate AR for 7 years. AVR was indicated for the progressively worsening AR and left ventricular function.

On admission, the patient’s blood pressure was 122/62 mmHg, and heart rate was 102 bpm with an irregular rhythm. Chest radiography showed a cardiothoracic ratio of 56%. Electrocardiography (ECG) revealed a heart rate of 100 bpm with atrial fibrillation. Transthoracic echocardiography revealed a QAV with a severe central AR jet due to incomplete coaptation. The left ventricular ejection fraction was 50%, without local asynergy. The left ventricular end-systolic and end-diastolic diameters were 46 mm and 62 mm, respectively, and the diameter of the aortic valve annulus was 23 mm. Coronary angiography revealed no significant coronary artery stenosis or anomalies. ECG-gated enhanced computed tomography was not performed, and no coronary ostium anomalies were detected preoperatively.

**Fig. 1:**
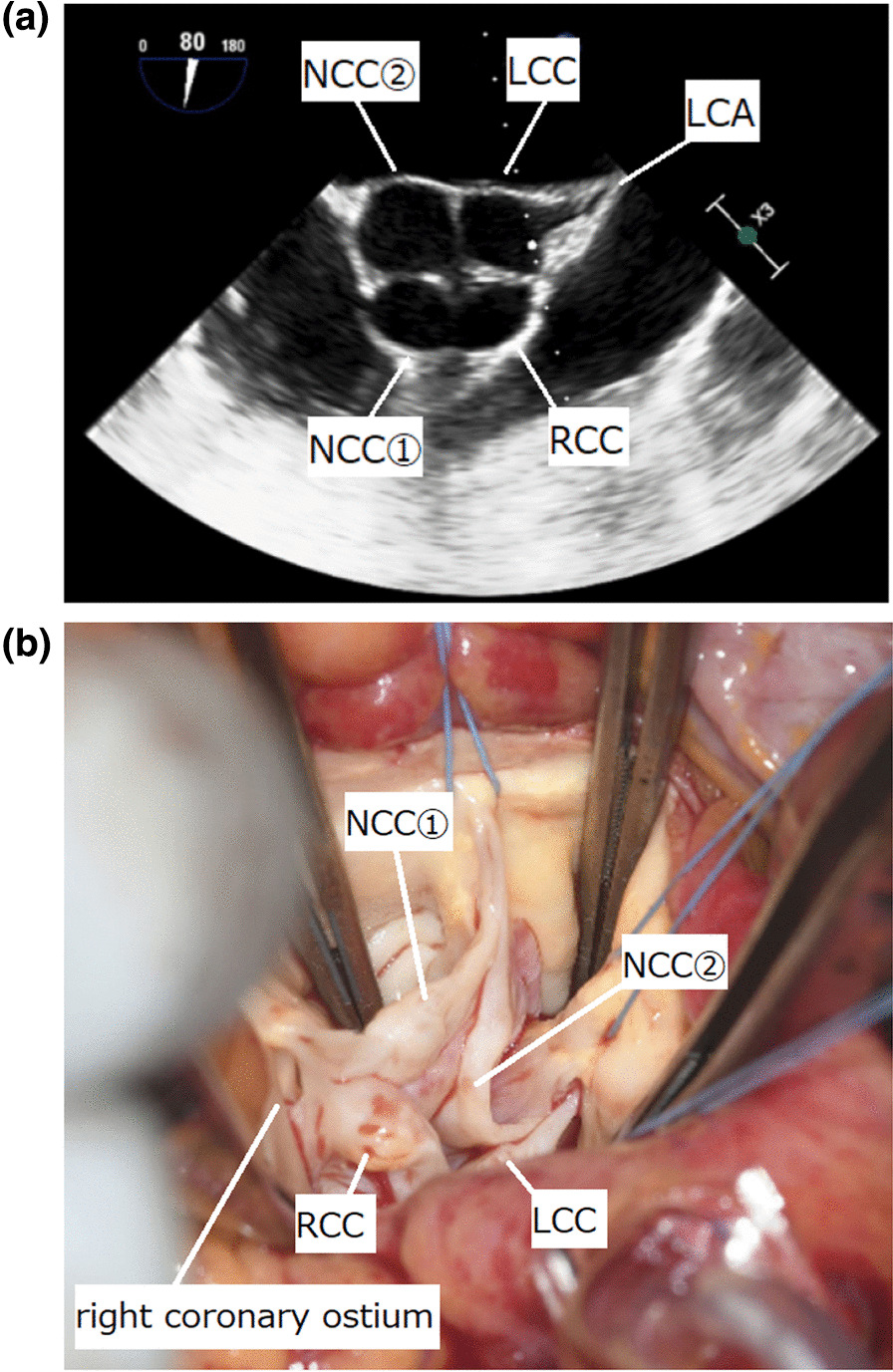
**a** Intraoperative transesophageal echocardiography showing almost equal-sized QAV (Hurwitz and Roberts classification type A). **b** Intraoperative photograph showing the QAV. The right coronary ostium is located adjacent to the commissure between the right coronary cusp and one of the two non-coronary cusps. LCA, left coronary artery; LCC, left coronary cusp; NCC, non-coronary cusp; QAV, quadricuspid aortic valve; RCC, right coronary cusp

**Fig. 2:**
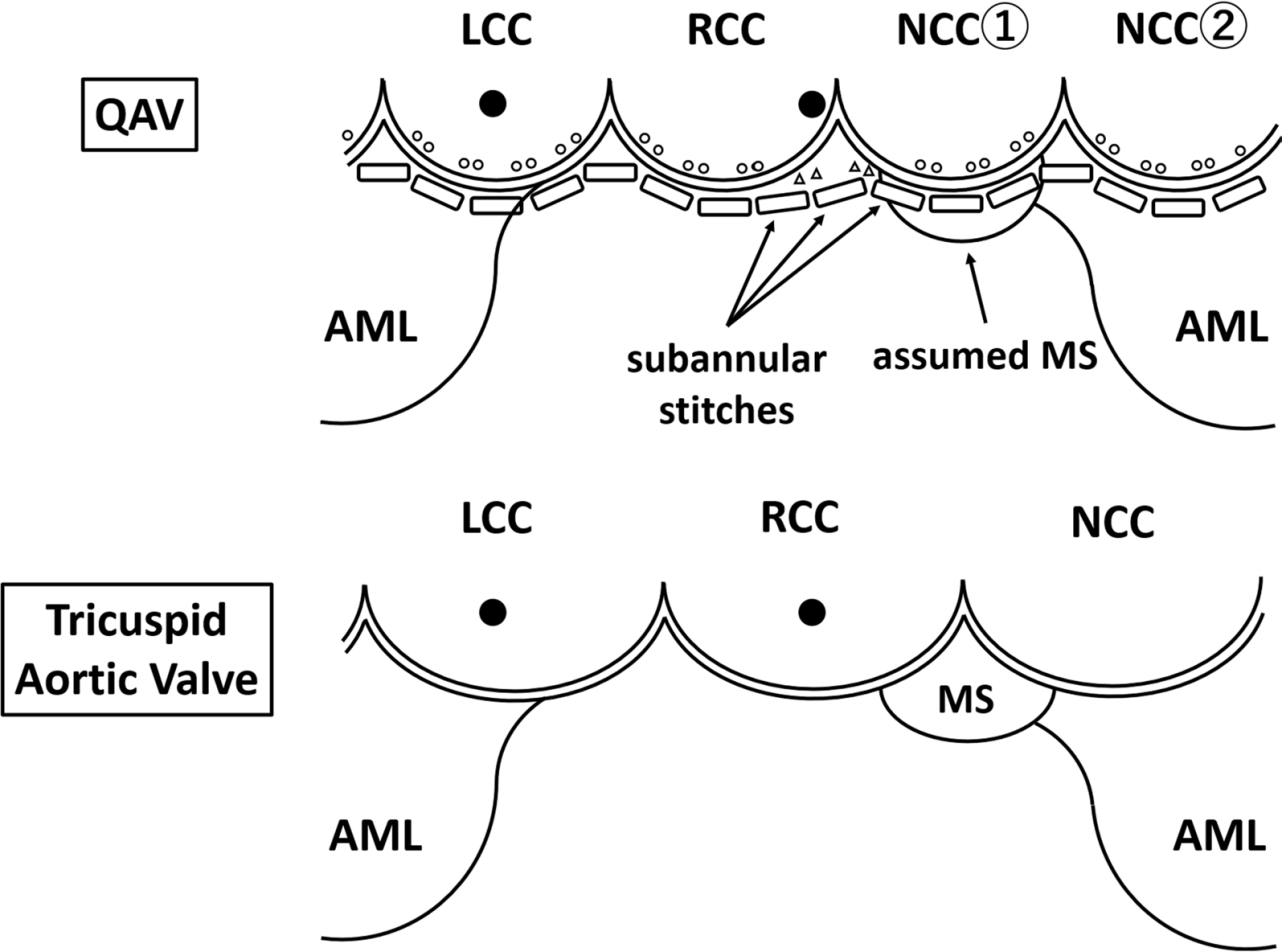
Operative schema of QAV in comparison with tricuspid aortic valve. The annular sutures are placed with pledgets on the ventricular side (circular symbols show exit points of stitches in a non-everting mattress fashion) and three stitches near the right coronary ostium are transitioned to the subannular ventricular myocardium (triangular symbols show exit points of stitches near the right coronary ostium). AML, anterior mitral leaflet; LCC, left coronary cusp; MS, membranous septum; NCC, non-coronary cusp; QAV, quadricuspid aortic valve; RCC, right coronary cusp

The patient underwent median sternotomy. The aortic valve had four cusps of almost equal size (Hurwitz and Roberts type A [1], Fig. 1). Macroscopically, partial calcification and thickening of the cusps were observed. The left coronary ostium was located in the middle of the left aortic sinus. Although the course of the right coronary artery was normal, the right coronary artery ostium was located slightly below the sinotubular junction and in close proximity to the commissure between the right coronary cusp and one of the two non-coronary cusps (Fig. 1). After excision of all cusps, the annular stitches were placed in a non-everting mattress fashion with pledgets on the ventricular side, and three stitches near the right coronary ostium were transitioned to the subannular ventricular myocardium to maintain the distance from the ostium (Fig. 2). We decided to use a biologic prosthesis, according to the patients’ desire. A 25-mm prosthetic sizer was able to pass through the annulus but mild resistance was noted. We selected a 23-mm Inspiris Resillia biologic prosthesis (Edwards Lifesciences, Irvine, California, United States of America), because an oversized prosthetic valve could potentially compress the right coronary ostium. In addition to AVR, pulmonary vein isolation using AtriCure (AtriCure, Mason, OH, USA) and left atrial appendage closure using AtriClip (AtriCure) was performed for atrial fibrillation.

The patient tolerated the procedure adequately, and postoperative echocardiography revealed normal prosthetic valve function without paravalvular leakage. Except for recurrent atrial fibrillation, the postoperative course was uneventful. The patient was discharged on postoperative day 17.

## Discussion

Coronary artery and coronary ostium anomalies, such as a single coronary artery and displacement of the left and right coronary ostia, are present in 2–10% of patients with QAV [2, 3, 6–8]. Despite a normal course through a coronary artery, the coronary ostium is occasionally positioned in close proximity to one of the commissures due to a QAV, as was the case with our patient. When performing AVR, care should be taken to avoid occluding or compressing the coronary ostium. Withana et al. reported AVR in a patient with a low origin of both the main coronary arteries and hypoplastic aortic annulus. Implantation of an oversized prosthetic valve resulted in severe stretching of the aortic annulus and the two coronary orifices, ultimately causing coronary ostial stenosis and occlusion [9]. Alsaddique et al. reported AVR in a patient with the left coronary ostium located close to the annulus. They placed everting mattress sutures with the pledgets on the aortic side; thus, the pledgets themselves partially obstructed the left coronary ostium and eventually required coronary artery bypass grafting [10]. In the case of an anomalous left circumflex artery originating from the right coronary artery and coursing behind the aortic annulus, the risk of ligation of this vessel and compression by the prosthetic valve must be considered during AVR [11]. In this case, we avoided obstructing the right coronary artery, which arose adjacent to the aortic annulus, through placing the annular sutures with the pledgets on the ventricular side. We used sutures that transitioned to the subannular ventricular myocardium in the area at closest proximity to the right coronary ostium as have been used by some surgeons on the commissures between the right coronary cusp and left coronary cusp to align the height of the stitches. However, the right coronary artery ostium still appeared to be in very close proximity to the valve sewing ring, potentially causing deformation, stenosis, or obstruction of the ostium. Since there was no prosthesis-patient mismatch in this case, a one-step smaller-sized prosthesis was selected to reduce the risk of coronary ostium obstruction as far as possible. We did not select the annular stitches placed from outside the aortic valve annulus because dissecting the Valsalva sinus and placing stitches under the origin of the right coronary artery was associated with an increased risk of injury of the right coronary ostium.

Suturing to the subannular ventricular myocardium is associated with a risk of causing a complete atrioventricular block, particularly in a patient with abnormal aortic valve development, as the anatomical relationship between the aortic annulus and the membranous septum (MS) is different. In fact, Pirundini et al. reported that complete atrioventricular block occurred after AVR for QAV, which had a small accessory cusp between the right and non-coronary cusp [12]. They also reported another case in which they avoided injury to the conduction system by suturing to a supra-annular position in the accessory cusp area. In these cases, the MS was meant to be located beneath the area of the small accessory cusp. Unlike their cases, however, the present case showed an almost equal-sized QAV with a normal course of the coronary system. The MS is generally connected to the right fibrous trigone; therefore, it was assumed that the MS was located on the right of the anterior mitral leaflet and beneath the area close to the nadir of one of the two non-coronary cusps. Thus, the subannular valve sutures around the commissure between the right coronary cusp and one of the two non-coronary cusps were effective in protecting the right coronary ostium, while avoiding complete atrioventricular block.

When coronary ostial occlusion is inevitable, even if every possible measure is taken, the addition of coronary artery bypass grafting should be considered.

## Conclusions

We report a case of AVR performed for a QAV that was associated with the right coronary artery ostium adjacent to one of the commissures. Careful selection of the size of the prosthesis and modification of the annular stitches are essential to prevent obstruction of the coronary ostium.

## Data Availability

The data are not available for public access due to patient privacy concerns but are available from the corresponding author upon reasonable request.

## Abbreviations

AR: Aortic regurgitation
AVR: Aortic valve replacement
ECG: Electrocardiography
MS: Membranous septum
QAV: Quadricuspid aortic valve
